# Protective efficacy of dietary natural antioxidants on microplastic particles-induced histopathological lesions in African catfish (*Clarias gariepinus*)

**DOI:** 10.1007/s11356-022-23789-w

**Published:** 2022-11-07

**Authors:** Alaa El-Din H. Sayed, Mervat N. Hana, Mohamed Hamed, Hany M. R. Abdel-Latif, Jae-Seong Lee, Hamdy A. M. Soliman

**Affiliations:** 1 Faculty of Science, Zoology Department, Assiut University, Assiut, 71516 Egypt; 2 Faculty of Science, Zoology Department, Al Azhar University (Assiut Branch), Assiut, 71524 Egypt; 3 Faculty of Veterinary Medicine, Department of Poultry and Fish Diseases, Alexandria University, Alexandria, Egypt; 4 Department of Biological Sciences, College of Science, Sungkyunkwan University, Suwon, 16419 South Korea; 5 Faculty of Science, Zoology Department, Sohag University, Sohag, 8562 Egypt

**Keywords:** Microplastics, Lycopene, Histology, Liver, Kidney

## Abstract

Microplastic particles (MPs) are a common environmental pollutant easily ingested by fish in aquaculture. The current study evaluated the protective efficacies of some antioxidant, e.g., lycopene, citric acid, and chlorella, against the toxic effects of MP ingestion by *Clarias gariepinus* using histopathological biomarkers. Five experimental groups were established, a control group receiving only a standard diet, a group exposed to 500 mg/kg MP concomitant with the standard diet, and three antioxidant groups exposed to MPs plus either lycopene (500 mg/kg), citric acid (30 g/kg), or chlorella (50 g/kg) in the standard diet. After 15 days, fish were sacrificed for histological and histochemical examinations. Histological analysis of the kidney for group 2 (fed 500 mg/kg MPs alone) revealed distributed tissue dissociation, regional glomerular hypertrophy or shrinkage, melanomacrophage accumulation, and expansion of Bowman’s space, while liver tissue exhibited dilation and rupture of the central vein wall, hemorrhage, cytoplasmic vacuolation, and cellular necrosis or apoptosis. Fish exposed to MPs also exhibited connective tissue fiber accumulation around renal blood vessels, renal tubules, the central hepatic vein, hepatic blood sinusoids, and serosal, muscle, and submucosal layers of the intestine. In addition, MP exposure reduced carbohydrate (mainly glycogen) contents in the brush borders and basement membranes of renal tubules, glomeruli, and intestinal tissues as well as in the cytoplasm of hepatocytes. These signs of renal, hepatic, and intestinal histopathology were fully or partially reversed by dietary lycopene, chlorella, or citric acid. Enhancing dietary antioxidants is an effective strategy for preventing MP toxicity in *Clarias gariepinus* in aquaculture.

## Introduction

Plastics are a group of synthetic and semisynthetic materials produced through the polymerization of monomers such as ethylene and propylene. Plastics are used extensively in manufacturing to reduce product weight, enhance strength, and provide transparency for display and other applications. The demand for plastics is growing, and many applications have demonstrably improved the quality of human life (Abarghouei et al. 2021). However, the production and ubiquitous use of nonbiodegradable plastics has resulted in widespread environmental contamination and progressive accumulation up the food chain from animals to humans. As emerging pollutants, microplastics (MPs) have attracted worldwide attention and recently become a research focus in aquatic environmental science. Terrigenous input was considered the main source of MP pollution (Cole et al. 2011). The total aquaculture production of the world is over 90 million tonnes, which exceed the capture production in 2020. In Egypt, aquaculture is currently the largest single source of fish, accounting for almost 65% of the domestic supply, of which over 99% is produced by privately owned farms (Kaleem and Sabi 2021). Microplastics are discovered and enriched in both farmed and captured species which regarded as one of the most important sources of high-quality protein. Both endogenous and exogenous factors like the use of fishing plastic products, factory farming facility and equipment, natural and synthetic feed, animal health products, aquaculture fortifier, and aquatic food additives make accumulation of microplastics easier. In addition, the safety of aquaculture products is closely related to human health because the residues of microplastics in fish lead to various potential hazards (Zhou et al. 2021).

MPs can pass and accumulate in fish through gill respiration or ingestion behavior, which has been detected in the gastrointestinal tract, gill, or circulatory system of fish (Zazouli et al. 2022). In general, larger MPs tend to accumulate in the digestive tract and be excreted more quickly, while smaller ones can be transported through the digestive tract to the circulatory system and more likely to remain in the fish (Kaposi et al. 2014). Some of the main effects that have been identified due to the ingestion of MPs include hindered predatory capacity, neurotoxicity, and reduced metabolic rate, as well as increased mortality rates (Vázquez-Rowe et al. 2021). There have been numerous reports on the histopathological effects of MPs on fish (da Costa Araújo et al. 2020; Espinosa et al. 2017; González-Doncel et al 2022; Jabeen et al. 2018; Karami et al. 2016; Liu et al 2022; Lu et al. 2016, 2022; Montero et al. 2022; Rainieri et al. 2018; Rochman et al. 2013; Xia et al. 2020; Yang et al. 2020; Yin et al. 2018; Yu et al. 2018), and it may be due to oxidative stress and inflammation (Hoseini et al. 2022a, b). Histopathological and histochemical examination methods provide sensitive indicators of cellular and tissue alterations within target organs such as the kidney and liver (Hamed et al. 2022b; Haredi et al. 2020; Sayed et al. 2022).

Lycopene, a red carotenoid present in many fruits and vegetables, is one of the most potent natural antioxidants owning to the abundance of conjugated dienes (Dai et al. 2021; Zhao et al. 2019, 2020a, b and 2021). In fact, lycopene has a singlet oxygen-quenching capacity twice that of β-carotene and 10 times higher than α-tocopherol (Sahin et al. 2014), suggesting utility as a dietary supplement to mitigate oxidative stress caused by toxicants, such as MPs. Similarly, citric acid (CA) may improve the antioxidant system via dietary route and has also been reported to increase the bioavailability of dietary minerals required for metabolic and catabolic reactions in aquaculture species when used as a feed additive (Zhang et al. 2016). Chlorella is one of the most commonly used microalga in aquaculture to improve nutrition and infectious disease resistance, reduce toxin levels (aquatic bioremediation), ameliorate stress, and decrease bacterial pathogenicity by disrupting quorum sensing (Nicula et al. 2018). Little is known about mitigation strategies using natural products against MP toxicity. Thus, these three agents may help mitigate the toxic effects of MPs in aquaculture.

African catfish (*Clarias gariepinus*) are widely distributed throughout Africa and are among the most valuable species for aquaculture (Gomaah and El Naggar 2004). Moreover, *Clarias gariepinus* is a major fish model used in toxicological studies (Sayed et al. 2016). The current study aimed to evaluate the protective efficacies of lycopene, CA, and chlorella against the toxic effects of MP ingestion by *C. gariepinus* using histopathological and histochemical biomarkers.

## 2. Materials and methods

### Chemicals

A raw MP powder (polyethylene microplastics; PE-MPs) was purchased from Toxemerge Pty Ltd. (Melbourne, Australia) and characterized by light and transmission electron microscopy (TEM) at the TEM Unit of Assiut University (Assiut, Egypt) using a JEOL JEM-1200 EX II system as described (Hamed et al. 2019b). Individual particles were irregularly shaped, but more than 90% were <100 nm in size. A stock solution of 2.5 g MPs/L was prepared in purified water (Milli-Q) according to the manufacturer’s instructions and stored under darkness at 4°C. For exposure experiments, the stock was sonicated and immediately diluted in fresh rearing water before each rearing water exchange. Lycopene, CA, and chlorella were purchased from Sigma-Aldrich (Cairo, Egypt) and added from freshly prepared stocks at the indicated concentrations.

### Fish exposure

*Clarias gariepinus* (weight 250–300g, length 20–25 cm, *n*= 150; 30 per group) were transported to the Fish Biology and Pollution Laboratory, Faculty of Science, Assiut University. Animals were deemed healthy and parasite-free according to Asian Fisheries Society-Fish Health Section (AFS-FHS) criteria (2007). The fish were kept in 100-L tanks containing aerated, dechlorinated tap water maintained at 20.5°C, pH = 7.4, and dissolved oxygen = 6.9 mg/L under a 12:12 light/dark photoperiod for 4 weeks before experimental treatments. During the experimental period, fish groups were feed the same commercial feed (3 % body weight) for the control or commercial diet combined with the different tested supplement and/or MPs dose for experimental groups as following: 1st group received a standard fish diet (control), 2nd group received a standard fish diet plus MPs (500 mg/kg according to Espinosa et al. 2019), 3rd group received a standard fish diet plus (MPs (500 mg/kg) **+** lycopene (500 mg/kg according to El-Gawad et al. 2019), 4th group received a standard fish diet plus (MPs (500 mg/kg) +CA (30g/kg according to Mahmoud et al. 2019), and 5th group received a standard fish diet plus (MPs (500 mg/kg) + chlorella (50g/kg according to Carneiro et al. 2020).

After 15 days of treatment, six fish from each group were randomly selected and anesthetized by ice to reduce stress during processing for histopathology and histochemistry (Hamed et al. 2019a).

### Histopathology and histochemistry

Small pieces of liver, kidney, and intestine were carefully collected, rinsed, and fixed in 10% neutral buffered formalin for at least 48 h, dehydrated in graded ethanol, cleared in xylene, and embedded in paraffin blocks. Sections were cut at 5 µm, dewaxed in xylene, and stained with Harris’s hematoxylin and eosin (H&E) to detect tissue degeneration and cell death, the Periodic acid Schiff(PAS) reaction to detect carbohydrate (mainly glycogen) content (Drury and Wallington 1980), or Masson’s trichrome method to detect fibrosis. Sections were visualized using an Olympus microscope (BX50F4, Olympus Optical Co., LTP, Japan).

### Ethics statement

Animal care and experimental protocols were approved by the Research Ethics Committee of the Faculty of Science, Assiut University.

## Results

The quantitative scoring of the histopathological lesions in the liver, kidney, and intestine are reported in Table 1.

**Table 1:** Semi-quantitative scoring of the histopathology in the liver, kidney, and intestine of *Clarias gariepinus* exposed to microplastic, lycopene, chlorella, and citric acid for 15-day exposure periods (*N* = 3)

Histopathologic lesion	C	MPs	MPs + lycopene	MPs + chlorella	MPs + citric acid
Liver					
Cytoplasmic vacuolation	−	+ + +	+ +	+ +	+ +
Hydropic degeneration	−	+ + +	+ +	+ +	+ +
Apoptotic cells	−	+ + +	+ +	+ +	+ +
Melanomacrophage aggregation	−	+	+ +	+ +	+ +
Hepatic necrosis	−	+ + +	+	−	−
Central vein dilation and rupture	−	+ + +	+	+ +	+ +
Hemorrhage	−	+	−	−	−
Infiltrations of inflammatory cells	−	+ +	+	+	+ +
Kidney					
Proliferation in the hemopoietic tissue	−	+ + +	+ +	+ +	+
Shrinkage of glomerulus	−	+ + +	+ +	+ +	+
Slight dilation of Bowman’s space	−	+ + +	+ +	+ +	+
Dilation of renal tubules	−	+ + +	+	+ +	+ +
Necrosis	−	+ + +	−	−	−
Melanomacrophages	−	+ + +	−	+ +	+ +
Dissociation	−	+ + +	+	+	+ +
Hypertrophy in the glomerulus	−	+ + +	+	+ +	−
Intestine					
Degeneration of the basement membrane of columnar cells	−	+ + +	+	+	+
Increase in the number of goblet cells	−	+ + +	−	−	+
Increase in the number of blood cells	−	+ + +	+ +	+ +	+
Increase in the number of folds	−	+ + +	+	−	+
Expansion at villi structure	−	+ + +	+ +	+ +	+ +
Degeneration of serosa with pyknotic nuclei	−	+ + +	+	+	+ +

Score: ( −) No alteration, ( +) mild alteration, (+ +) moderate alteration, (+ + +) severe alteration

*C* control, *MPs* microplastics

### Prevention of MP-induced liver damage by lycopene, chlorella, and citric acid

**Fig. 1:**
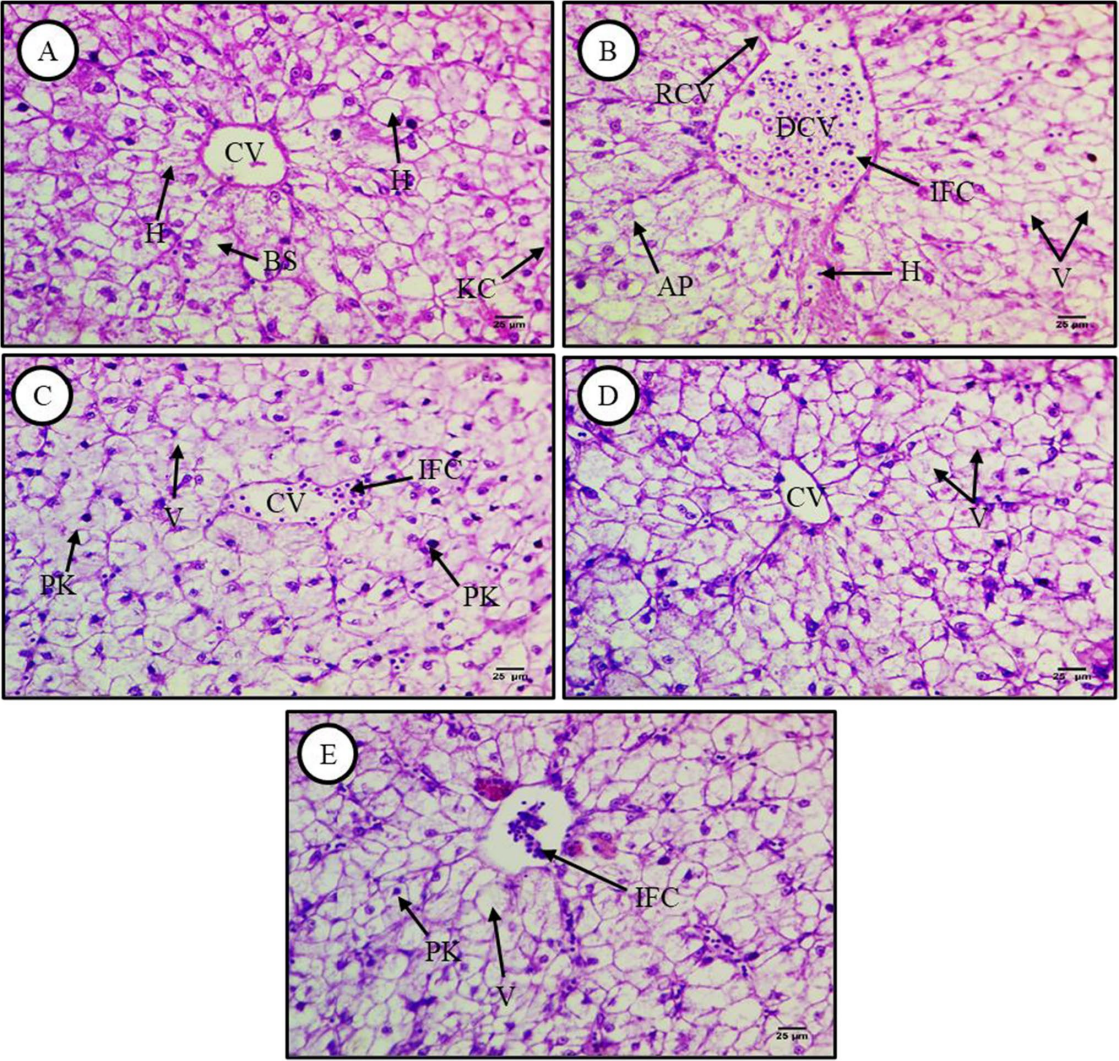
Hematoxylin and eosin (H&E)-stained liver sections from control and treated fish (× 400). **A** Sections from control fish showing the typical hepatic microstructure. Labeled structures are blood sinusoids (BS), the central vein (CV), hepatocytes (H), and Küpffer cells (KC). **B** Liver section from an MP-exposed fish (500 mg/kg) showing infiltration of inflammatory cells (IFC), apoptotic cells (AP), vaculations (V), rupture and dilatation of the central vein wall (RCV), and hemorrhage (H). **C** Liver section from a fish exposed to MPs (500 mg/kg) plus lycopene (500 mg/kg) showing reversal of histopathology except that some cells presented with pyknotic nuclei (PK) and vacuoles (V), and the tissue was infiltrated with inflammatory cells (IFCs). **D** Liver section from a fish exposed to MPs and chlorella (50 g/kg) showing reversal of histopathology except for some cell vacuolation (V). **E** Liver section from a fish exposed to MPs and citric acid (30 g/kg) showing reversal of histopathology except for some cell vacuolation (V) and aggregation of melanomacrophages (Mm)

Hematoxylin and eosin (H&E)-stained sections of the liver from *C. gariepinus* fed a normal diet (control group), exposed to MP in addition to normal diet feeding (MP group), or exposed to MP plus lycopene, MP plus chlorella, or MPs plus CA in addition to the normal diet feeding for 15 days are presented in Fig. 1A-E, respectively. The liver tissue of control *C. gariepinus* exhibited typical histological features, including regularly arranged wedge-shaped hepatocytes with central nuclei surrounding bile canaliculi, blood sinusoids containing Küpffer cells, and a prominent intact central vein (Fig. 1A). In contrast, sections from *C. gariepinus* exposed to MPs (500 mg/kg) showed various degrees of injury, including cellular necrosis and apoptosis, cytoplasmic vacuolation, dilatation and rupture of the central vein wall, and hemorrhage (Fig. 1B). These pathological signs were largely reversed by dietary supplementation with lycopene, as sections from the MP plus lycopene group demonstrated normal hepatocyte morphology and arrangement, although the number of the Küpffer cells was markedly higher and the number of melanomacrophages lower than in control sections. In addition, some cells exhibited pyknotic nuclei and vacuolation, while some tissue regions showed infiltration of inflammatory cells (Fig. 1C). Liver sections from fish exposed to MP plus chlorella also exhibited a relatively well-preserved tissue structure, although some cellular vacuolation was observed (Fig. 1D). Similarly, hepatic tissues from fish exposed to MPs plus CA showed normal histological structures but with some cellular vacuolation as well as enhanced melanomacrophage aggregation (Fig. 1E).

**Fig. 2:**
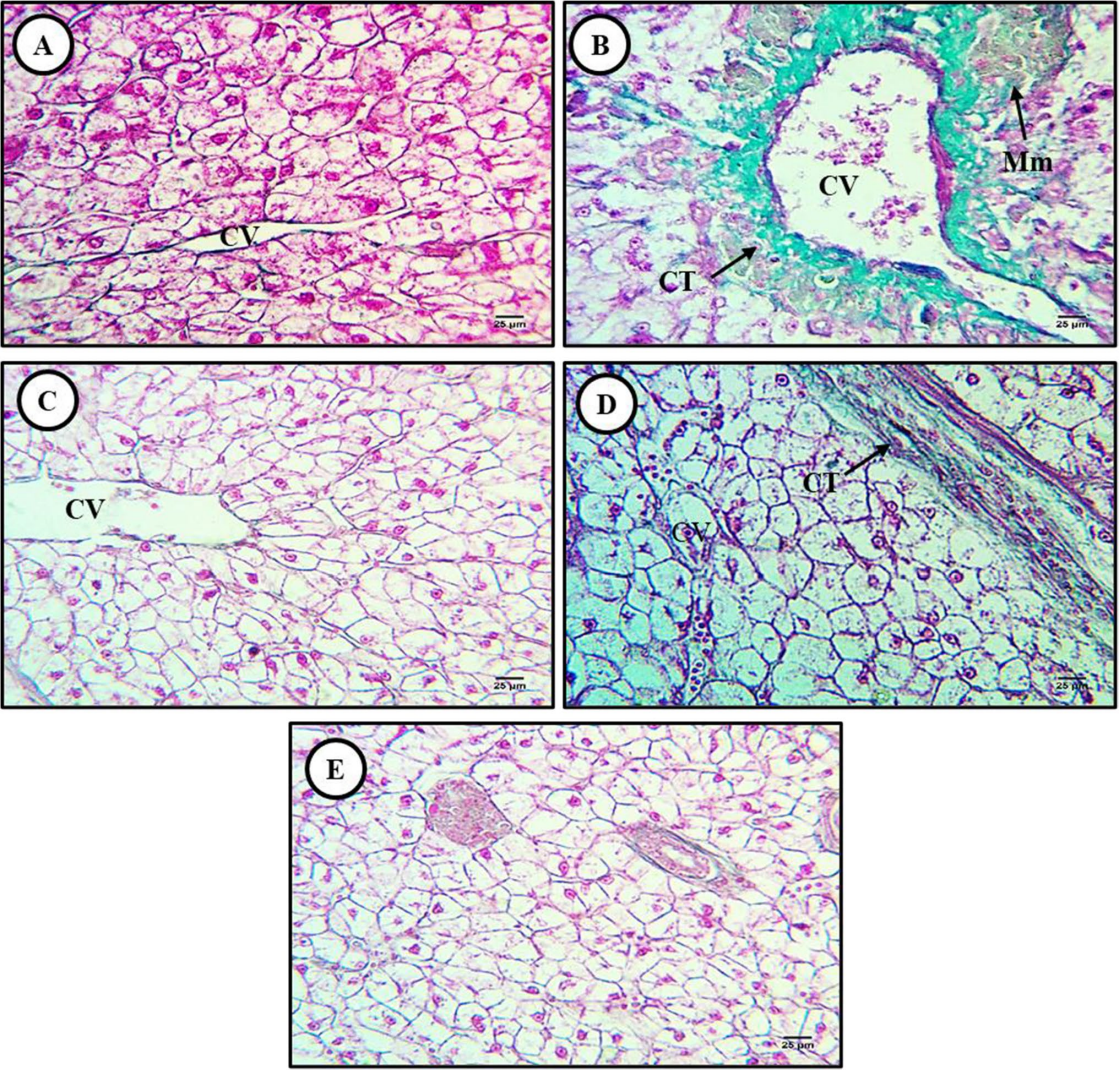
Masson’s trichrome-stained liver sections from control and treated fish (× 400). **A** Section from a control fish showing the typical hepatic tissue structure, including a prominent central vein (CV) surrounded by a small number of connective tissue fibers. **B** Liver section from an MP-exposed fish (500 mg/kg) showing a marked increase in connective tissue fibers (CT) around the central vein and blood sinusoids (fibrosis) as well as accumulation of melanomacrophages (Mm) beside the central vein. **C**–**E** Liver sections from fish exposed to MPs plus **C** lycopene (500 mg/kg), **D** chlorella (50 g/kg), or **E** citric acid (30 g/kg) showing reversal of fibrosis around the central vein and blood vessels

In liver sections from control catfish, Masson’s trichrome staining revealed sparsely distributed connective tissue (collagen) fibers around the hepatic central vein (Fig. 2A), while sections from MP-exposed fish exhibited substantial connective tissue fiber accumulation around the central vein and blood sinusoids (Fig. 2B). This fibrosis was completely reversed by cotreatment with lycopene and CA and moderately by cotreatment with chlorella (Fig. 2C–E).

**Fig. 3:**
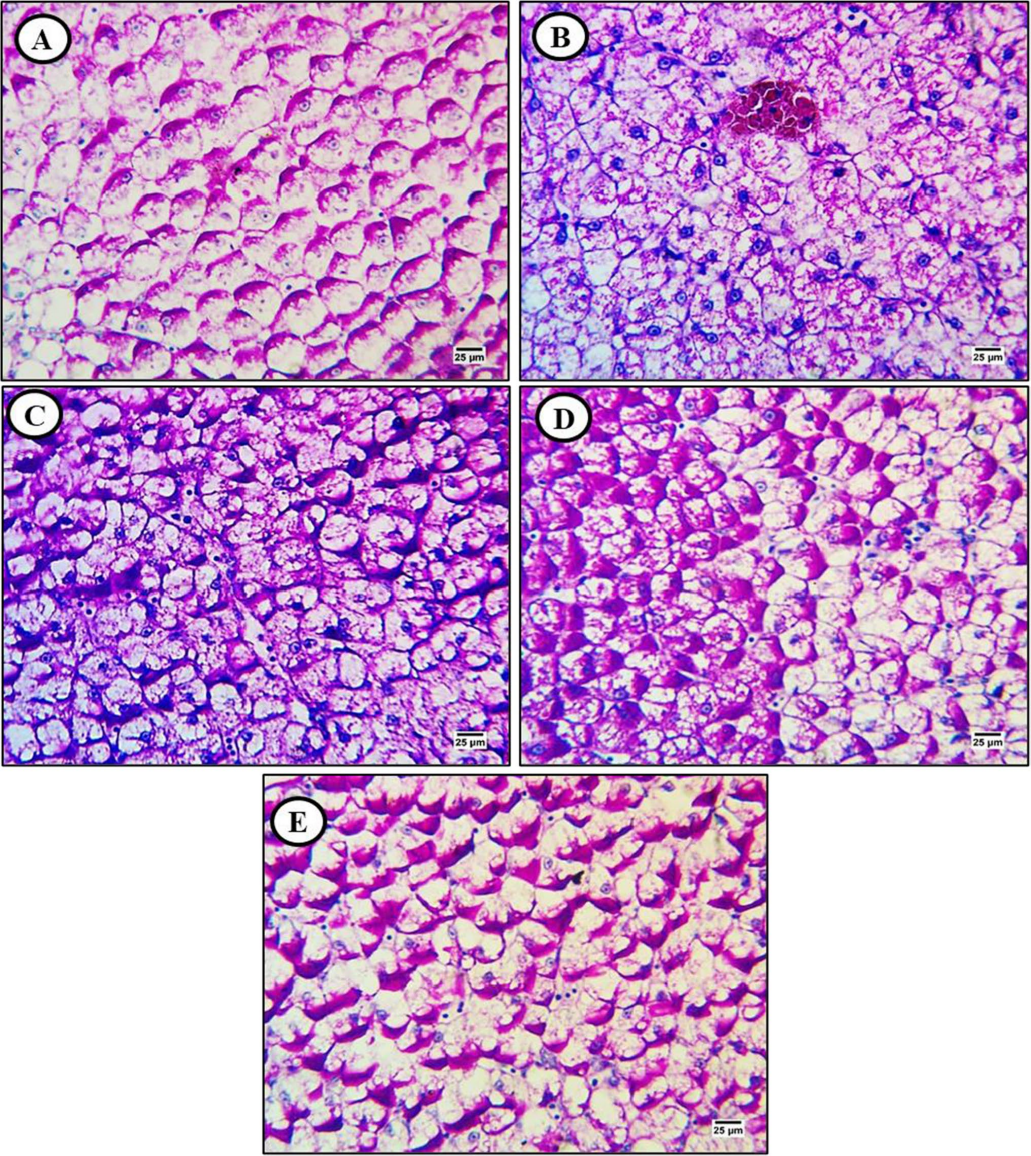
Periodic acid Schiff (PAS)-strained liver sections from control and treated fish (× 400). **A** Liver section from a control fish showing the typical distribution of glycogen in hepatocyte cytoplasm. **B** Liver section from a MP-exposed fish (500 mg/kg) showing glycogen depletion. **C** Liver section from a fish exposed to MP plus lycopene (500 mg/kg) showing reversal of glycogen depletion. **D** Liver **s**ection from a fish exposed to MPs plus chlorella (50 g/kg) showing partial reversal of glycogen depletion. **E** Liver section from a fish exposed to MPs plus citric acid (30 g/kg) showing moderate reversal of glycogen depletion

In the liver, the PAS technique primarily stains glycogen stores within hepatocytes as verified by PAS staining with diastase pretreatment (Fig. 3A). The PAS technique revealed distinct glycogen distributions among treatment groups. In sections from MP-exposed fish, PAS staining revealed a substantial reduction in hepatocellular glycogen content (Fig. 3B) that was almost completed reversed by lycopene, partially reversed by chlorella, and modestly reversed by CA (Fig. 3C–E)**.**

### Prevention of MP-induced kidney damage by lycopene, chlorella, and citric acid

**Fig. 4:**
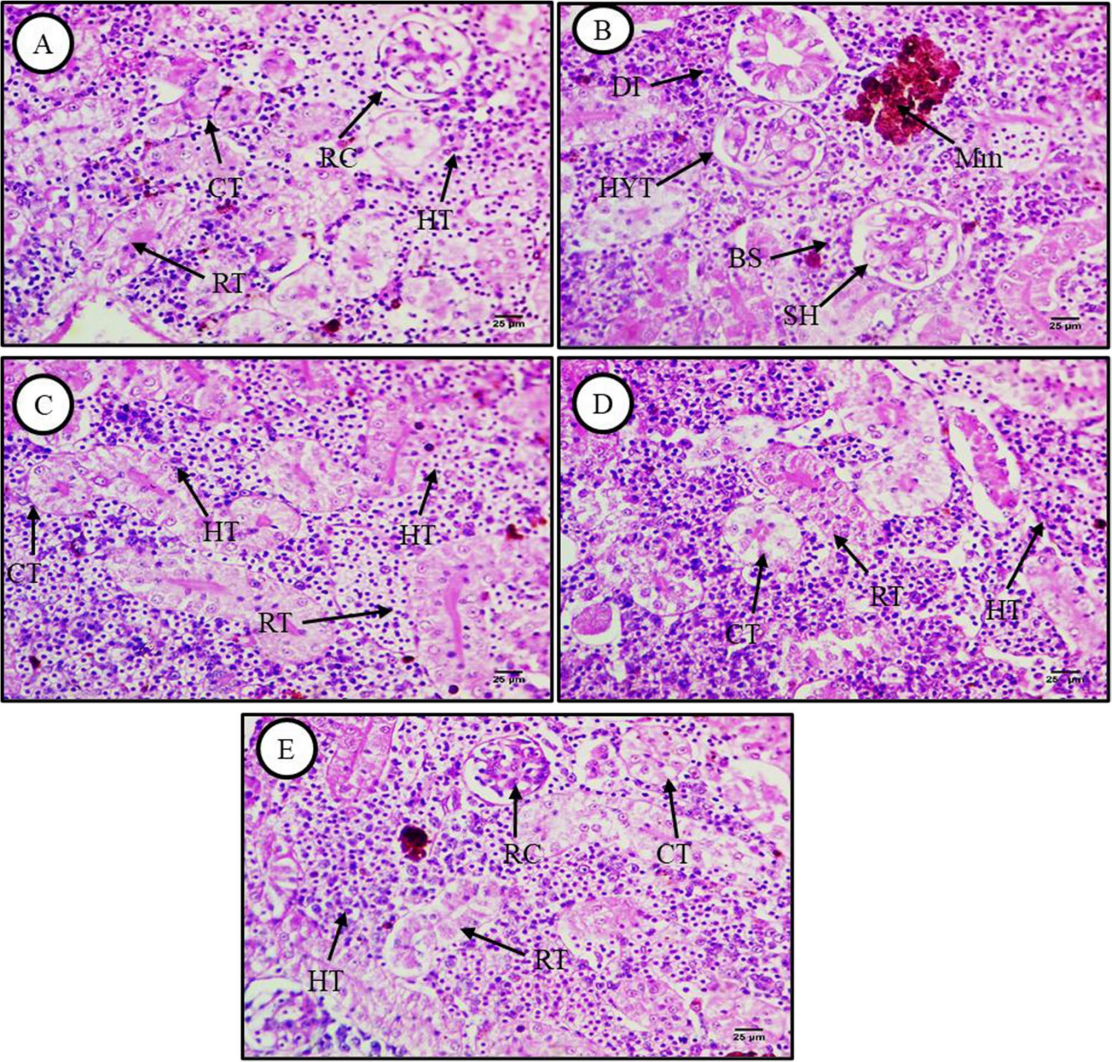
Hematoxylin and eosin (H&E)-stained transverse kidney sections from control and treated fish (× 400). **A** Kidney section from a control fish showing the typical renal microstructure, including renal corpuscles (RC), renal tubules (RT), collecting tubules (CT), and hemopoietic tissue (HT). **B** Kidney section from a fish exposed to MPs (500 mg/kg) showing tissue dissociation (DI), hypertrophy of glomeruli (HYT), melanomacrophage accumulation (Mm), shrinkage of glomeruli (SH), and expansion of Bowman’s space (BS). **C** Kidney section from a fish exposed to MPs and lycopene (500 mg/kg) showing improved renal microstructure. **D** Kidney section from a fish exposed to MPs plus chlorella (50 g/kg) showing reversal of renal degeneration except for some residual renal tubule dissociation (DI). **E** Kidney section from a fish exposed to MPs plus citric acid (30 g/kg) showing improved renal histology but residual hydropic degeneration (D)

**Fig. 5:**
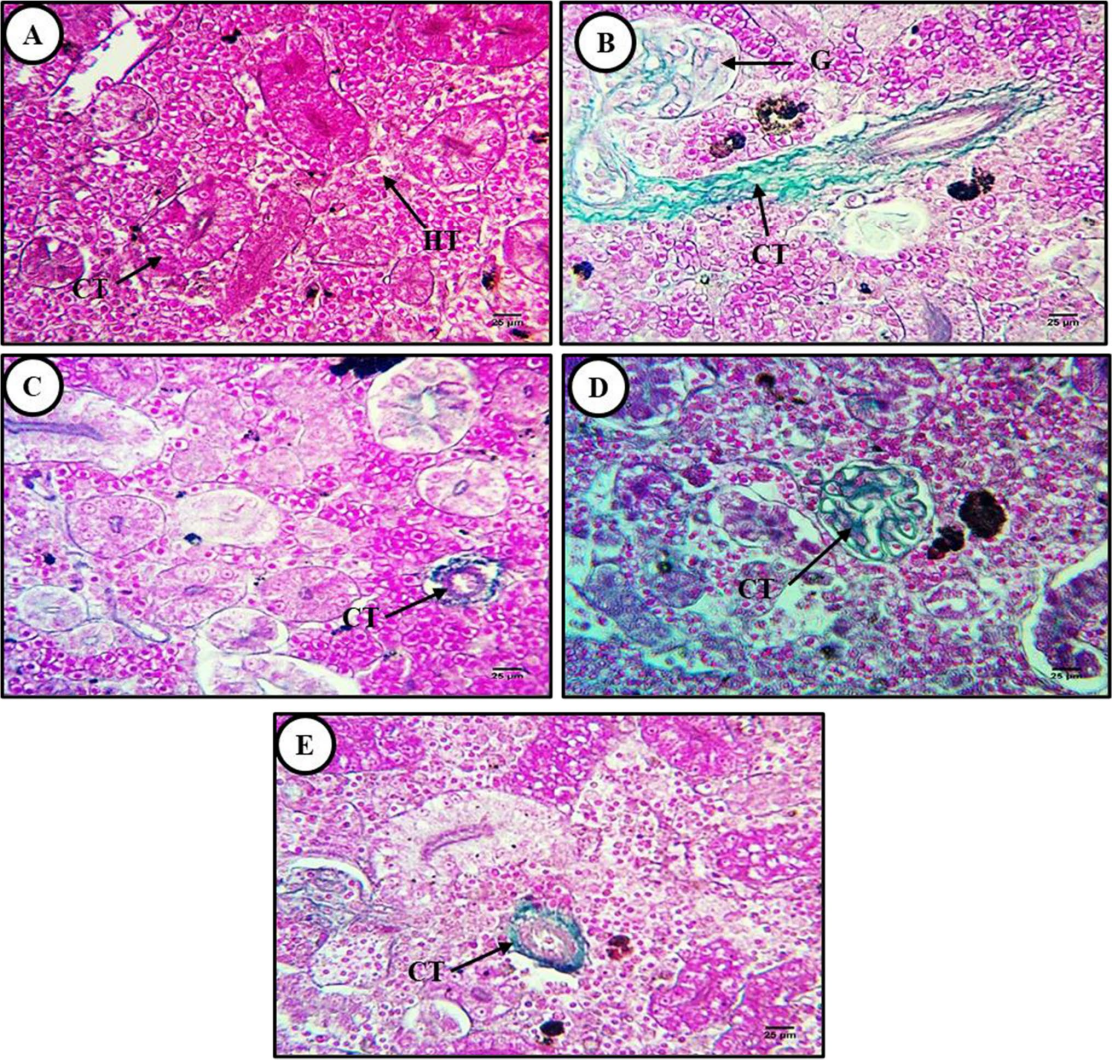
Masson’s trichrome-stained transverse sections from control and treated fish kidney (Masson’s trichrome × 400). **A** Section from a control fish showing sparse connective tissue fibers (CT) around renal corpuscles (RC) and renal tubules (RT). **B** Section from a fish exposed to MPs (500 mg/kg) showing large accumulations of connective tissue fibers (CT) around blood vessels and renal tubules as well as substantial thickening of blood vessels walls. **C**–**E** Sections from fish exposed to MPs plus **C** lycopene (500 mg/kg), **D** chlorella (50 g/kg), or **E** citric acid (30 g/kg) showing reversal of fibrosis

**Fig. 6:**
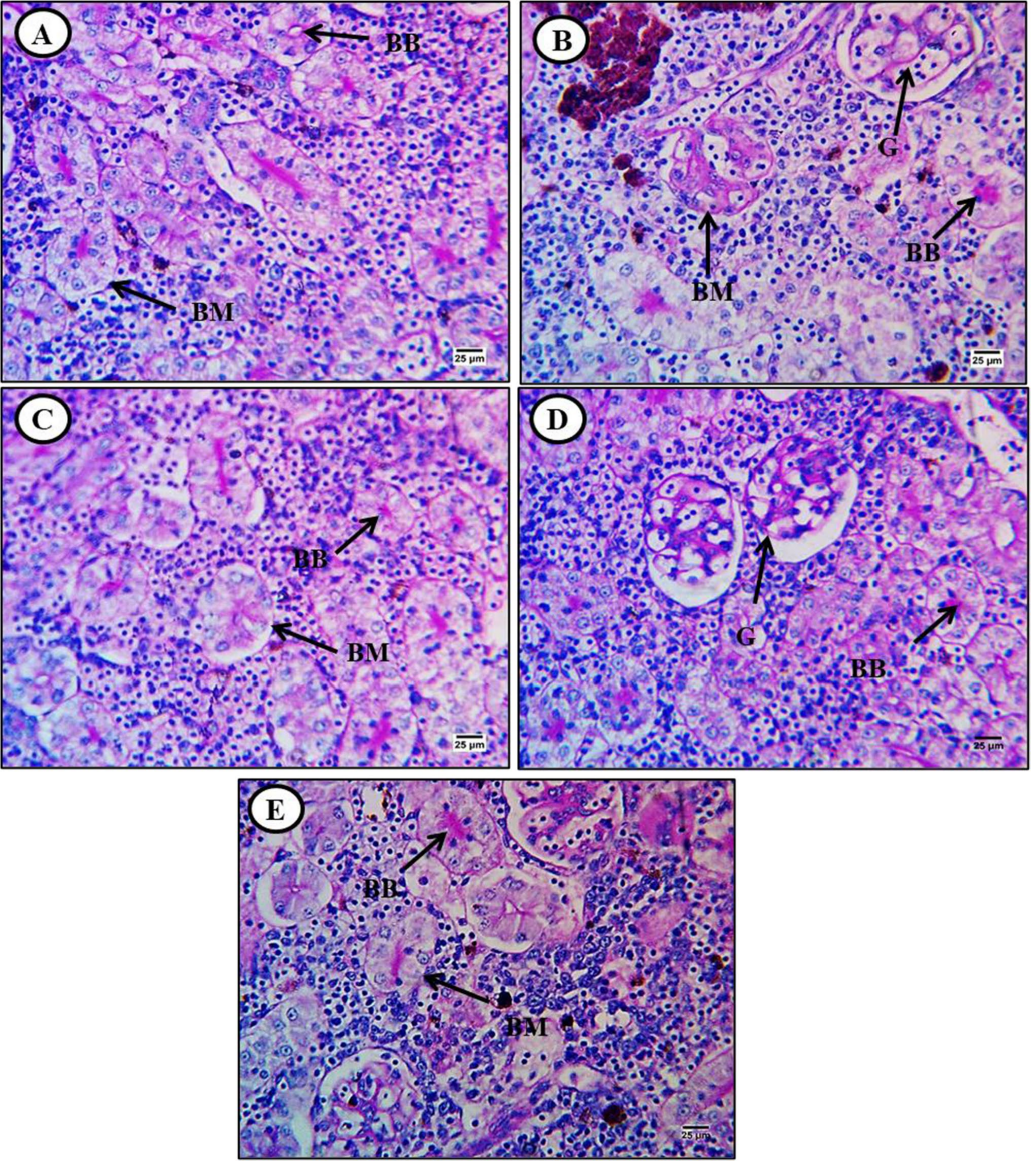
Periodic acid Schiff-stained transverse sections of kidney from control and treated fish (× 400). **A** Kidney section from a control fish showing extensive PAS reactivity (glycogen) in the brush border (BB) and basement membrane (BM) of renal tubules. **B** Kidney section from a fish exposed to MPs (500 mg/kg) showing glycogen depletion in the brush border (BB) and basement membrane (BM) of renal tubules and glomeruli (G). **C** Kidney section from a fish exposed to MPs plus lycopene (500 mg/kg) showing reversal of glycogen depletion in the basement membrane (BM) and brush border (BB). **D** Kidney section from a fish exposed to MPs plus chlorella (50 g/kg) showing moderate reversal of glycogen depletion. **E** Kidney section from a fish exposed to MPs plus citric acid (30 g/kg) showing moderate reversal of glycogen depletion in glomeruli, basement membranes, and hemopoietic tissue

Histological sections of kidney from *C. gariepinus* exposed to these same five treatment conditions for 15 days are presented in Figs. 4A, 5, and 6E. Hematoxylin and eosin-stained sections from control fish (Fig. 4A) revealed the typical arrangement of glomeruli, Bowman’s capsules, tubular lumens, blood vessels, hemopoietic tissues, and intrarenal spaces, while HE-stained sections from MP-exposed fish demonstrated dissociation, glomerular hypertrophy and shrinkage, melanomacrophage accumulation, and expansion of Bowman’s spaces. Like liver tissue, these histopathological abnormalities were reversed by lycopene (500 mg/kg) (Fig. 4C). Administration of chlorella (50 g) also largely reversed these changes, although some renal tubule dissociation was still observed (Fig. 4D). Similarly, these abnormalities were largely reversed by CA (30 g), although some renal tubules showed hydropic degeneration (Fig. 4E).

Also similar to liver tissues, Masson’s trichrome staining revealed few connective tissue fibers around renal corpuscles and renal tubules in sections from control fish (Fig. 5A) but substantial accumulation around renal blood vessels and tubules as well as vessel wall thickening in sections from MP-exposed fish (Fig. 5B). The accumulation of connective tissue fibers was reversed by coadministration of lycopene, chlorella, or CA (Fig. 5C–E).

Kidney sections from control fish showed robust PAS reactivity in brush borders and basement membranes of renal tubules as well as within hematopoietic tissue (Fig. 6A), while carbohydrate staining was markedly reduced in sections from MP-exposed fish (Fig. 6B). Dietary supplementation with lycopene substantially reversed the MP-induced decrease in basement membrane, brush border, and hematopoietic tissue carbohydrate content (Fig. 6C), while chlorella (Fig. 6D) and CA (Fig. 6E) had more modest effects.

### Prevention of MP-induced intestinal damage by lycopene, chlorella, and citric acid

**Fig. 7:**
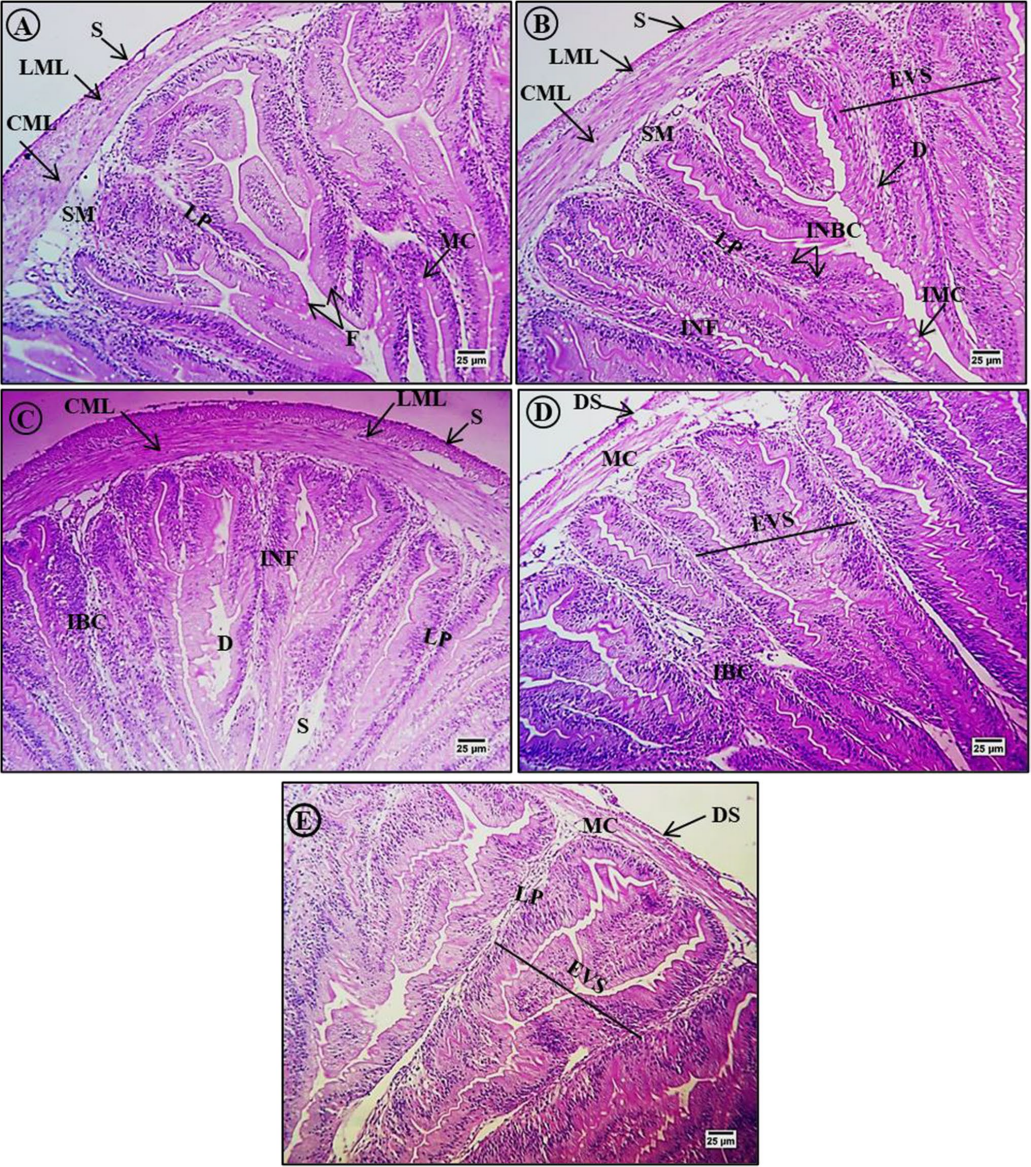
Hematoxylin and eosin (H&E)-stained sections of intestine from control and treated fish (× 400). **A** Section of the intestine from a control fish showing the typical layered structure including mucosal layer folds (F), lamina propria (LP), submucosa (SM), circular muscle layer (CML), longitudinal muscle layer (LML), and serosa (S). **B** Intestinal section from a fish exposed to MPs (500 mg/kg) showing degeneration (D), increased numbers of mucus cells (IMC), blood cells (INBC), and folds (INF) and expansion of villi structures (EVS). **C** Intestinal section from a fish exposed to MPs plus lycopene (500 mg/kg) showing partial reversal of microstructural changes but with residual increases in the number of blood cells (IBC) and folds (INF) as well as some degeneration (D) and larger spaces (S) at the base of villi. **D** Intestinal section from a fish exposed to MP plus chlorella (50 g/kg) showing partial reversal of intestine microstructure disruption except for a residual increase in the number of blood cells (IBC), some degeneration of serosa (DS), and expansion of villi structures (EVS). **E** Intestinal section from a fish exposed to MP plus citric acid (30 g/kg) showing partial reversal of microstructure disruption except for some degeneration of the serosa (DS) and expansion of villi structures (EVS)

**Fig. 8:**
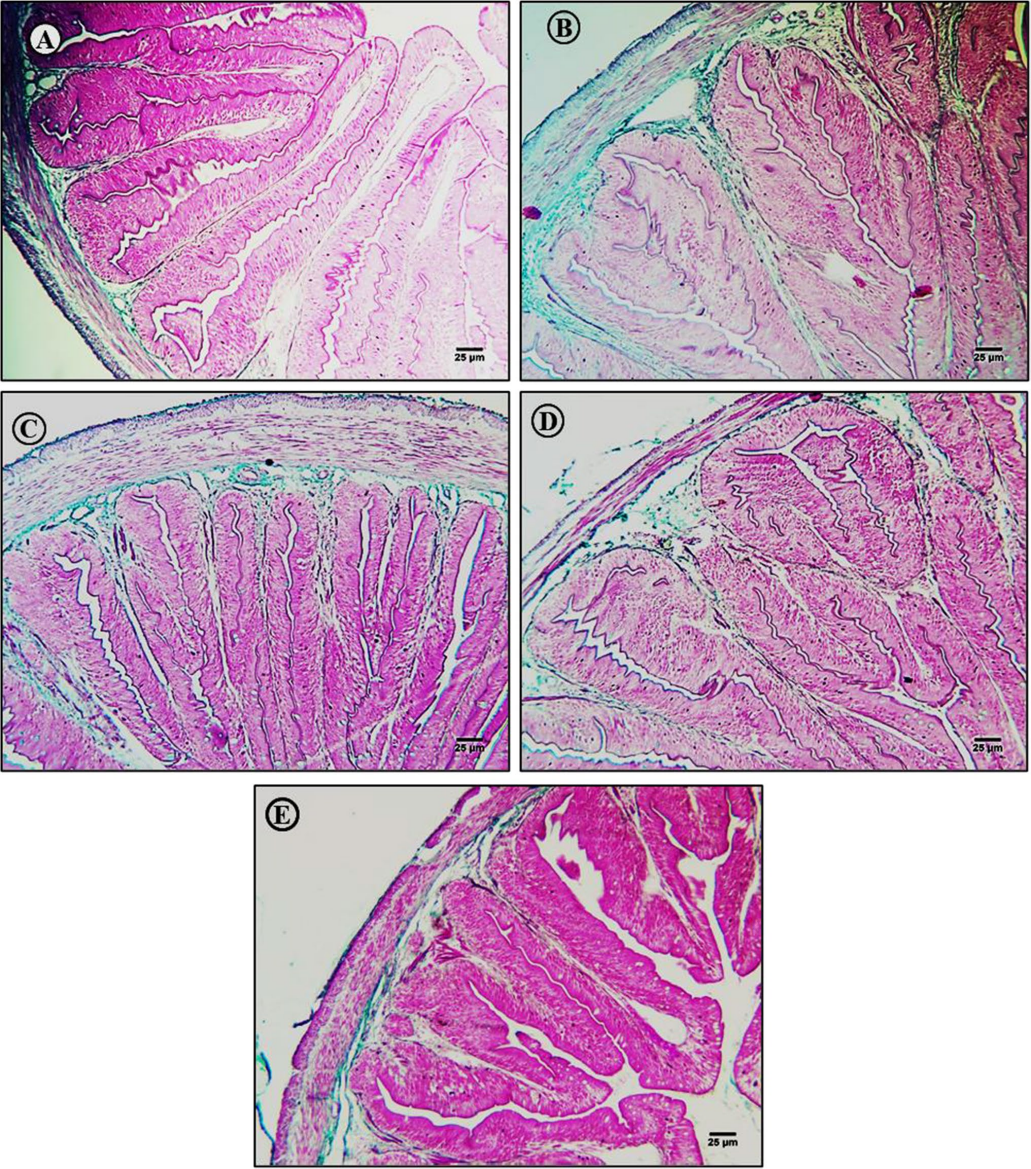
Masson’s trichrome-stained intestinal sections from control and treated fish (× 400). **A** Section from a control fish intestine showing the typical layered structure and distribution of connective tissue fibers in the submucosal layer. **B** Section of intestine from a fish exposed to MPs (500 mg/kg) showing substantial accumulation of connective tissue fibers in serosal, muscle, and submucosal layers. **C**–**E** Sections of intestine from fish exposed to MPs plus **C** lycopene (500 mg/kg), **D** chlorella (50 g/kg), and **E** citric acid (30 g/kg) showing reversal of fibrosis in the submucosa layer

**Fig. 9:**
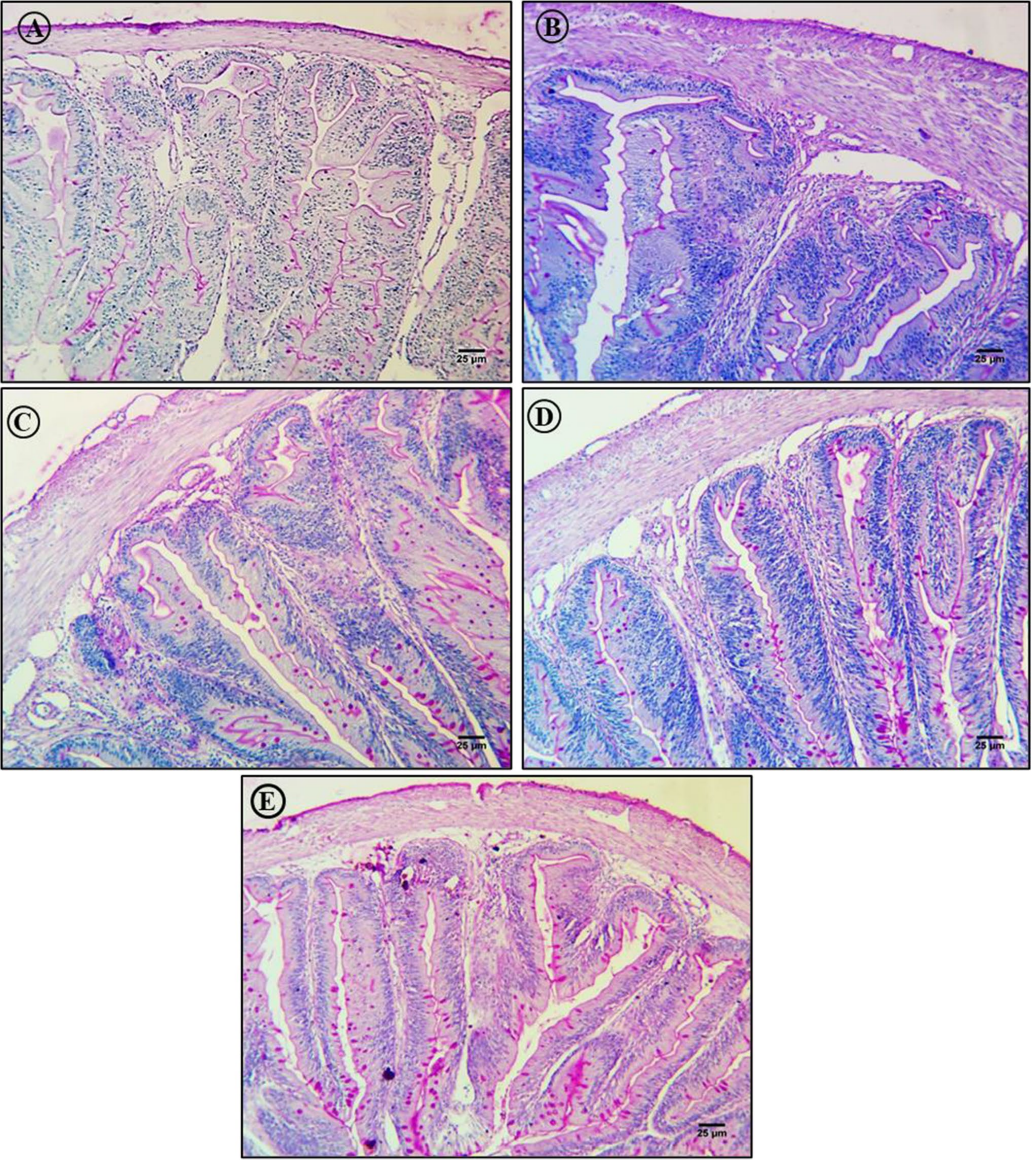
Period acid Schiff-stained intestinal sections from control and treated fish (× 400). **A** Intestinal section from a control fish showing substantial amounts of cytoplasmic glycogen. **B** Intestinal section from a fish exposed to MPs (500 mg/kg) showing depletion of cytoplasmic glycogen. **C** Intestinal section from a fish exposed to MPs plus lycopene (500 mg/kg) showing reversal of glycogen depletion. **D** Intestinal section from a fish exposed to MP plus chlorella (50 g/kg) showing partial reversal of glycogen depletion. **E** Intestinal section from a fish exposed to MPs and citric acid (30 g/kg) showing moderate reversal of glycogen depletion

Histological sections of intestine from *C. gariepinus* exposed to these same 5 treatment conditions for 15 days are presented in Figs. 7A, 8, and 9. H&E staining of sections from control group *C. gariepinus* revealed the typical layered structure of the intestinal wall. The mucosal layer was composed of long columnar cells and numerous goblet cells (mucus-producing cells) with centrally placed nuclei, and the luminal side was boardered by finger-like villi. Also as expected, the submucosal layer was thin, projected into mucosal folds, and was composed mainly of loose connective tissue (the lamina propria) with numerous collagen fibers and blood cells. The muscle layer consisted of inner thick circular muscle layers and outer thin longitudinal muscle layers, while the serosa consisted of a peritoneal cell layer and blood capillaries (Fig. 7A). In contrast, intestinal sections from MP-exposed *C. gariepinus* revealed degeneration of basement membrane columnar cells as well as an increase in the number of goblet cells, blood cells, and folds, as well as expansion at villi structures (Fig. 7B). These changes were largely revered by lycopene, although the numbers of blood cells and folds were still elevated compared to control tissue sections (Fig. 7C). Administration of chlorella also largely reversed these changes, although again there was an increase in the number of blood cells, expansion of villi structures, and numerous serosal cells with pyknotic nuclei (Fig. 7D). Similarly, intestinal sections from fish exposed to MPs plus CA exhibited a normal layered structure except for serosal degeneration with pyknotic nuclei and expansion of villi structures (Fig. 7E).

Masson’s trichrome stain revealed only sparse connective tissue fiber accumulation in the intestinal submucosa layer of control fish (Fig. 8A), while MP-exposed fish exhibited large accumulations of connective tissue fiber in serosal, muscle, and submucosal layers (Fig. 8B). Administration of lycopene, chlorella, or CA reversed fiber accumulation, especially in the submucosal layer (Fig. 8C–E).

Finally, PAS staining revealed punctate glycogen stores (as verified by PAS staining with and without diastase pretreatment) distributed throughout intestinal layers (Fig. 9A). Glycogen content was markedly reduced by MP exposure (Fig. 9B), an effect substantially reversed by lycopene and more moderately by chlorella and CA (Fig. 9C–E)**.**

## Discussion

The present study provides evidence supporting the efficacies of lycopene, citric acid, and chlorella against MP-induced liver, kidney, and intestinal damage in African catfish. Histological analysis of kidney sections from MP-exposed catfish revealed microstructural breakdown, glomerular hypertrophy, melanomacrophage accumulation, glomerular shrinkage, and expansion of Bowman’s spaces. Fish exposed to MPs also exhibited connective tissue fiber accumulation around renal blood vessels and renal tubules as well as thickened blood vessel walls and a marked decrease in the carbohydrate (mainly glycogen) content within the brush borders and basement membranes of renal tubules and glomeruli. Previous reports have documented MP-induced expansion and congestion of glomerular capillaries, increased glomerular size, glomerular atrophy, vacuolation of glomerular cells, expansion of Bowman’s spaces, inflammatory cell infiltration, shrinkage and convolution of tubules, widening of intertubular spaces, and signs of fatty tubules among other pathogenic changes (Espinosa et al. 2017; González-Doncel et al. 2022; Hamed et al. 2022a; Hodkovicova et al. 2021; Meng et al 2022; Zhu et al. 2020). These changes may stem from increased intrarenal pressure associated with MP-induced congestion and concomitant mechanical stress on glomerular, capillary, and tubular walls (Ichimura et al. 2013), from cellular oxidative stress, or from both pathomechanisms.

Liver tissue from MP-exposed catfish also exhibited signs of histological damage, including dilatation and rupture of the central vein wall, hemorrhage, cytoplasmic vacuolation, hepatocellular apoptosis, and accumulation of connective tissue fibers around the central vein and blood sinusoids (fibrosis). In addition, MP-exposed liver demonstrated a substantial decline in cytoplasmic glycogen. As the liver is a major storage organ for glucose (as glycogen), MPs may have marked detrimental effects on systemic metabolism. Several previous studies have also reported signs of hepatic damage following MP ingestion, including cellular vacuolation, immunocyte infiltration, passive hyperemia, hydropic degeneration, dilatation and congestion of blood sinusoids, loss of parenchymal organization, focal necrosis, severe deformation of hepatocytes, and pyknotic nuclei (da Costa Araújo et al. 2020; Espinosa et al. 2019; Jabeen et al. 2018; Hoseini et al. 2022a, b; Hu et al. 2022; Karami et al. 2016; Liao et al. 2022; Liu et al. 2022; Lu et al. 2016, 2022; Rainieri et al. 2018; Rochman et al. 2013; Xia et al. 2020; Yang et al. 2020; Yin et al. 2018; Yu et al. 2018). These pathological changes are also observed in response to prooxidant poisoning, and in liver diseases associated with oxidative stress (Sayed et al. 2016, 2021a, b).

Exposure to MP also induced various reactive changes in intestinal structure, such as increased numbers of (mucus-producing) goblet cells, blood cells, and mucosal layer folds, and expansion of villi structures, as well as signs of tissue damage, including degeneration of basement membrane columnar cells and fibrosis of serosal, muscle, and submucosa layers. Similar to kidney and liver tissues, MP exposure also reduced tissue carbohydrate content. Previous studies have reported similar structural and pathological changes following MP exposure, including shortening, erosion, and swelling of villi; vacuolation, swelling, and blebbing of enterocytes, focal necrosis of enterocytes and mucosal epithelial cells, disruption of the epithelial boundary, complete detachment of the epithelium and lamina propria, infiltration of leukocytes, degeneration of the basement membrane, goblet cell hypertrophy, atrophy of the submucosa, pyknotic nuclei, hemorrhage, and vacuolation of mucosal cells (Ahrendt et al. 2020; Chen et al. 2022; Espinosa et al. 2019; Guo et al. 2022; Iheanacho and Odo 2020; Jabeen et al. 2018; Liao et al. 2022; Lei et al. 2018; Lu et al. 2016; Montero et al. 2022; Pedà et al. 2016; Qiao et al. 2019a; Song et al. 2019; Xia et al. 2018b; Zhang et al. 2022; Zhu et al. 2020; Zuo et al. 2022). Exposure to MPs also enhanced the number of mucosal neutrophils as revealed by PAS straining (Limonta et al. 2019) and markedly reduced goblet cell coverage as well as gut mucus volume, indicating epithelial layer damage (Qiao et al. 2019b). Sıkdokur et al. (2020) observed that both goblet cell numbers and PAS reaction intensity decreased in intestinal tissue exposed to microplastics and mercury. Microplastic particles also dose-dependently enhanced collagen deposition as evidenced by Masson’s trichrome staining (Hamed et al. 2020; Li et al. 2020).

Dietary supplementation with lycopene, CA, or Chlorella largely prevented these MP-induced morphological and histochemical abnormalities in renal, intestinal, and hepatic tissues. Chlorella contains chlorella growth factor, which promotes cellular proliferation and tissue repair and also activates the immune system to facilitate the removal of dead cells (Ratucoreh and Retnoaji 2018). Dietary chlorella was also reported to increase goblet cell numbers as evidenced by PAS and immunostaining (Ratucoreh and Retnoaji 2018) and to reduce histopathological alterations (Zahran et al. 2019), possibly due to the presence of bioactive components with documented antioxidant, antibacterial, antiviral, and (or) anti-inflammatory properties such as carotenoids, alkaloids, flavonoids, glycosides, phenols, lignins, saponins, amino acids, and carbohydrates (Yanuhar et al. 2020).

In this study, CA was applied at 30 g/kg, while a previous study found that reversal of lipopolysaccharide (LPS)-induced liver damage was more pronounced at 2 g/kg (Abdel-SalamOmar et al. 2014). Also, CA at 200 mg/kg reduced hepatic tissue damage and vacuolation of hepatic cells induced by the organophosphate insecticide malathion (Abdel-Salam et al. 2016). Thus, the optimal dose appears to depend on the specific toxin and model organism, so the mild effects observed in this study may reflect suboptimal dosing. Further, the protective effects of CA are likely to involve both antioxidant and bioenergetic actions. CA supplementation also alleviated soybean meal‐induced qualitative alterations in mucus layers, prevented intestinal lesions, and mitigated intestinal oxidative damage as evidenced by reduced accumulation of the membrane peroxidation product MDA and greater cellular antioxidative capacity (Chen et al. 2018).

Numerous studies have shown that lycopene can protect animals against environmental toxins such as herbicides and insecticides, likely by reducing oxidative stress as lycopene was reported to be the most efficient carotenoid singlet O_2_ quencher (Di Mascio et al. 1989). In rats, lycopene administration reduced renal toxicity of the insecticide deltamethrin by quenching free radicals and other antioxidant effects (El-Saad 2011). El-Gerbed (2014) reported that lycopene also reduced deltamethrin-induced collagen deposition in tubules and glomeruli, while effects on perivascular fibrosis and hyalinization of glomeruli and proximal tubular epithelium were not examined. Treatment of rats exposed to the herbicide atrazine protected against adrenal damage in part by restoring antioxidant defenses and by attenuating NF-kB/caspase-3-dependent apoptosis (Abass et al. 2016). Lycopene was also reported to have greater efficacy for reducing thioacetamide-induced fibrosis and fibroblast proliferation than other carotenoids (Akdemir et al. 2016). In addition, dietary lycopene prevented atrazine-induced renal pathology by modulating CYP450 homeostasis and the nuclear xenobiotic receptor response (Xia et al. 2018a). Dietary lycopene also prevented hepatopancreas damage induced by the insecticide endosulfan (although mild to moderate fatty changes were still present) (Hussein et al. 2019). Zhao et al. (2020a, b) found that lycopene (LYC) prevented sulfamethoxazole (SMZ)-induced oxidative damage by restoring redox balance, by promoting immune homeostasis and CYP450 activation, and by inactivating the caspase/Bcl2 apoptotic pathway. In addition, lycopene reduced histological damage to the liver and kidney induced by the acetochlor-based herbicide Harness® by stabilizing the plasma membrane, thereby preserving the structural integrity of cells and facilitating repair of tissue damage (Sayed et al. 2021a).

## Conclusions

Fish exposed to MPs exhibited histopathological changes in different organs. Dietary administration of lycopene, CA, or chlorella ameliorated the histological damage in liver, kidney, and intestine of African catfish. Hence, dietary supplementation of these agents may be an effective strategy to prevent fish death in aquaculture farms due to MP contamination.

## Data Availability

All data generated or analyzed during this study are included in the research article.
